# Similar Cost Savings of Bundled Payment Initiatives Applied to Lower Extremity Total Joint Arthroplasty Can Be Achieved Applying Both Models 2 and 3

**DOI:** 10.1007/s11420-017-9571-7

**Published:** 2017-08-18

**Authors:** Allyson Alfonso, Lorraine Hutzler, Bill Robb, Chad Beste, André Blom, Joseph Bosco

**Affiliations:** 1 NYU Hospital for Joint Diseases, 301 East 17th Street, Suite 1402, New York, NY 10003 USA; 2 Illinois Bone & Joint Institute, 900 Rand Road, Suite 300, Des Plaines, IL 60016 USA

**Keywords:** BPCI, total joint replacement, cost savings, institutional comparisons, post acute care, readmissions

## Abstract

**Background:**

In an effort to control cost and increase value, Medicare is transitioning from fee-for-service to value-based alternative payment models (APMs). The Bundled Payments for Care Improvement (BPCI) initiative represents one such voluntary APM. BPCI offers four different bundling options: model 1 covers all Diagnosis Related Groups (DRGs) and Models 2–4 cover 48 clinical episodes, including 186 separate DRGs.

**Questions/Purposes:**

The purpose of this investigation is to analyze and compare the cost savings achieved by two different BPCI program participants, provider A and provider B, enrolled in different models of BPCI (Models 2 and 3) for lower extremity joint replacements (LEJRs).

**Methods:**

We analyzed the BPCI cost savings for Medicare Severity-Diagnosis Related Groups (MS-DRGs) 469 and 470 (lower extremity joint replacement) of two different BPCI program participants. One (provider A) participated in Model 2 while the other (provider B) participated in Model 3. Retrospective payments were based upon savings generated by decreased actual expenses reconciled against target pricing for the episode of care in Models 2 and 3.

**Results:**

The Model 2 participant reduced the average cost of all episodes by 18.45%, with all of the savings occurring in the post acute phase. The Model 3 participant reduced episode costs by 16.73%.

**Conclusion:**

Both BPCI providers achieved similar cost savings despite participating in different BPCI models. These cost savings all occurred in the post acute setting. The Model 2 provider achieved post acute savings through decreasing overall discharges to institutional post acute care (PAC) providers and decreasing readmissions, while the Model 3 provider decreased costs largely by decreasing the LOS for the institutional PAC providers and decreasing readmissions.

**Electronic supplementary material:**

The online version of this article (doi:10.1007/s11420-017-9571-7) contains supplementary material, which is available to authorized users.

## Introduction

The percentage of US gross domestic product spent on health care is projected to increase from 17.8 to 20.1% from 2015 to 2025 [1]. In an effort to control this growth rate and improve the quality of care, the federal government is transitioning from traditional fee-for-service payment arrangements to value-based alternative payment models (APMs). The Centers for Medicare and Medicaid Services (CMS) established a goal of transitioning 30% of US health care payments to APM by 2016, increasing to 50% by 2018 [2]. The Bundled Payments for Care Improvement (BPCI) is one such APM, which has broad applications to orthopedic surgery.

In 2013, CMS established the BPCI program. It was designed to control health care costs and increase the value (outcomes or quality/cost) of health care [3, 4]. BPCI is composed of four model bundled payment types. Model 1 covers all Medicare Severity-Diagnosis Related Groups (MS-DRGs) for the acute inpatient hospital stay only. Models 2–4 cover 48 clinical episodes, including 186 separate DRGs. Models 2 and 3 are retrospective bundled payments where providers receive fee-for-service (FFS) payments that are later reconciled against target prices for episodes of care. Model 2 includes hospital and physician inpatient and 90-day post discharge services, while model 3 includes 90-day post discharge services only. Conversely, Model 4 is a prospective bundled payment made to the hospital that is meant to fund all hospital services, including health care providers, during the entire inpatient stay [3–5].

The purpose of this investigation is to analyze and compare the cost savings achieved by two different BPCI program participants, provider A and provider B, enrolled in different models of BPCI (Models 2 and 3) for lower extremity joint replacements (LEJRs).

## Methods

We compared two BPCI program participants, providers A and B, for LEJRs (MS-DRG codes 469 and 470, major joint replacement or reattachment of lower extremity with and without major complications or comorbidities (MCC)). The two providers were similar in that they were located in major urban centers and both were affiliated with major academic medical centers, but different in that provider A was one urban, tertiary orthopedic hospital with 40 surgeons providing LEJR care and provider B was an independent orthopedic practice of 44 surgeons providing LEJR care at 16 hospitals supporting five area health care systems. Provider A participated in Model 2 and provider B participated in Model 3. Baseline data was from DRGs 469 and 470 from 2009 to 2012 for each provider. The BPCI performance period began in October 2013 for Model 2 and December 2013 for Model 3 and ending in September of 2015 for both providers. We utilized CMS’s reconciliation cost analysis for each provider to determine episode cost savings and to determine where and how these savings were achieved.

Both Models 2 and 3 represent a retrospective bundled payment. The episode of care for Model 2 begins 72 hours before admission and continues up to 90 days after discharge. The episode of care for Model 3 begins at initiation of post-acute care services including home health care (HHC) and institutional post-acute care (PAC) providers (skilled nursing facility (SNF), inpatient rehabilitation facility (IRF), or long-term care hospital (LTCH) and continues up to 90 days post discharge. Services included in the 90-day post discharge period for both model types are the post-acute care services listed above, as well as, hospital outpatient services, independent outpatient therapy services, clinical laboratory services, durable medical equipment, and Medicare Part B drugs and pharmacy services [3, 6].

## Results

**Fig. 1:**
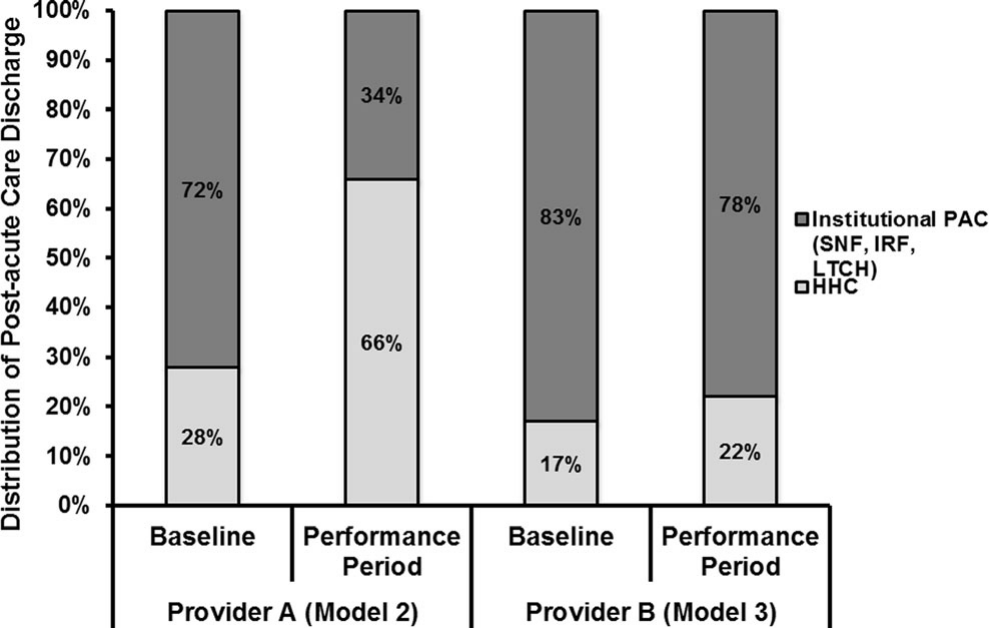
Distribution of post acute care discharge before (baseline) and after (performance period) implementation of BPCI Models 2 and 3.

**Fig. 2:**
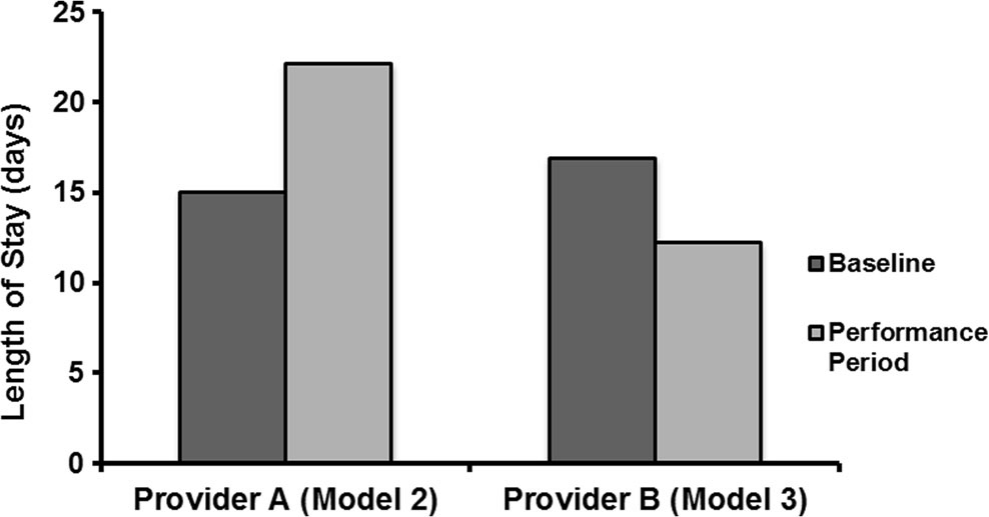
Post discharge institutional PAC provider length of stay in days before (baseline) and after (performance period) implementation of BPCI Models 2 and 3.

Baseline data from 2009 to 2012 yielded 1905 episodes for provider A (Model 2) and 5410 episodes for provider B (Model 3) for MS-DRGs 469 and 470. Provider A had 1680 episodes in its performance period and provider B reported 3298 episodes in its performance period. After initiating the BPCI models, the Model 2 participant increased the percentage of discharge to HHC from 28 to 66% while decreasing the discharge percentage to institutional PAC providers (SNF, IRF, or LTCH) from 72 to 34%. The Model 3 participant increased the discharge percentage to HHC from 17 to 22% and decreased the discharge percentage to institutional PAC providers from 83 to 78% (Fig. 1). Institutional PAC provider length of stay (LOS) was increased by the Model 2 participant from 15.0 to 22.1 days and reduced by the Model 3 participant from 16.9 to 12.2 days (Fig. 2). The Model 2 participant increased the length of engagement for HHC from 15.7 to 22.3 days, while the model 3 participant reduced the length of stay from 19.0 to 15.1 days.

**Fig. 3:**
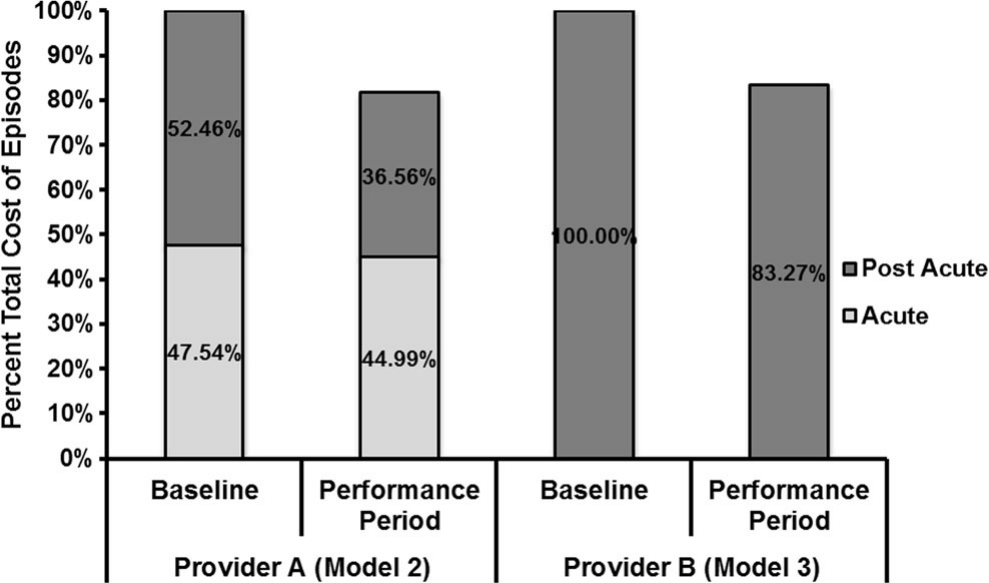
Percent total cost of episodes relative to baseline (100%), before (baseline), and after (performance period) implementation of BPCI models 2 (acute and post acute) and 3 (post acute).

The average cost for all episodes was reduced by 18.45% (both hospital and post-acute) for provider A (Model 2) and 16.73% for provider B (Model 3) (Fig. 3). Readmission rates decreased for both institutions. Provider A’s readmissions decreased from 13.0 to 6.4%, while provider B’s decreased from 12.8 to 9.2%.

## Discussion

We analyzed and compared episode-based cost savings of LEJR bundles between two similar institutions employing different BPCI models. Both providers achieved a similar percentage of cost savings (18.45 and 16.73%), and these cost savings all occurred during the post discharge phase of the episode. However, each provider achieved these cost savings in a unique manner. Provider A (Model 2) achieved savings by decreasing post discharge admissions to institutional PAC providers (SNF, IRF, or LTCH) from 72 to 34%, increasing usage of HHC, and reducing readmission rates from 13.0 to 6.4%. Provider B (Model 3) achieved the majority of its cost savings, not by decreasing discharges to institutional PAC providers, but instead by decreasing the institutional PAC provider LOS from 16.9 to 12.2 days, and by decreasing readmission rates from 12.8 to 9.2%. Decreasing LOS for inpatient facilities is an important savings driver, as institutional PAC providers are paid on a per diem basis. This means that decreasing LOS days by 50% translates into a 50% savings. In contrast, engagements for HHC are reimbursed on a case rate basis (a flat fee for the entire engagement no matter how many days). This means any change in the length of engagement of HHC episodes does not affect costs.

The limitations of our study include those associated with the small sample size of two providers, which differed in region, as sole representations for each model type. High geographic variation in hospital charges for total joint arthroplasty have been noted [11], as well as individual differences that may be present among physician preferences in post acute discharge facilities and availability of facilities in each region. The goal of this study, however, was not to evaluate Model 2 in contrast to Model 3, although such comprehensive studies to evaluate best bundle practices may be completed in the future. It is important to note that these cost savings were observed in the first year of the BPCI contract, and further savings are anticipated with the increased familiarity of both program objectives and outcomes among participating providers.

For both providers and both BPCI models, savings were achieved in the post-acute care discharge costs, which included readmission costs and post acute care costs. The Lewin Group analyzed the year 1 results of BPCI Models 2–4. They reported that all cost savings for Model 2 occurred in the post-acute care period. Their analysis demonstrated that index hospitalization inpatient costs actually rose slightly, from $14,256 to $15,663 for BPCI Model 2. They reported an average percent savings in surgical orthopedic excluding spine episodes of 13% using Model 2 [4].

Slover et al. reported that post acute discharge to HHC, even if this requires a longer acute hospital stay, is more cost effective than discharge to inpatient facilities [7]. This is partly due to the length of stay and rate of readmission for patients discharged to institutional PAC providers [8]. Medicare reimburses both SNF and IRF stays on a per diem rate, thus providing an economic disincentive for these institutions to decrease their LOS. However, as provider B demonstrated, decreasing post acute LOS is an effective way to decrease episode-based costs. Additionally, readmissions are an important cost driver in bundled arrangements. Controlling readmission rates is essential in order to remain financially viable in a setting of bundled payments. The margins for total knee arthroplasty (TKA) and total hip arthroplasty (THA) are estimated to be 4.3 and 2.8%, respectively [9]. Considering these relatively small margins, controlling the expense of readmissions becomes critical for a financially successful bundle [10].

Implementation of LEJR BPCI Models 2 and 3 by two providers reduced the cost of the episode of care. Total bundled costs and savings as a percentage of the episode are similar in both BPCI model type 2 and 3. Additionally, all the cost savings achieved in both models occurred in the post acute setting. Provider B (model 3) achieved its post discharge cost savings by both decreasing the length of stay in institutional PAC providers (SNF, IRF, or LTCH) and reducing readmissions, while provider A achieved its savings by reducing the overall utilization of institutional PAC providers and decreasing the readmission rate. The ability of these two providers to incorporate geographic and hospital-orthopedic practice integration differences in BPCI program designs, yet achieve similar cost savings, is vital to the program’s nationwide success.

The success of the voluntary BPCI program has led, at least in part, to CMS implementing the mandatory Comprehensive Care for Joint Replacement (CJR) program. The major differences between CJR and BPCI are in reporting requirements and reconciliation methods. CMS believes the changes made will encourage the creation of financial relationships between participant hospitals and other providers in order to coordinate quality and efficiency goals [12]. The model began on April 1, 2016. This mandatory bundle affects 67 metropolitan statistical areas and includes 33% of all LEJR performed in the USA. The implementation of CJR is contributing to CMS’s stated goal of having 50% of all Medicare fee-for-service payments made via alternative payment models by 2018 [13].

Our results contribute to the assessment of BPCI and future implications of CJR. Despite variations in practice geography, hospital orthopedic practice integration, and post discharge rehabilitation care patterns, cost savings can be achieved through different methods that improve quality of care for patients such as discharge to HHC versus institutional PAC providers and decreases in both readmission rates and length of stay in hospital. Future research includes longitudinal studies beyond 1 year, comparative analysis between models to evaluate best bundle practices, and assessment of programs stemming from BPCI like CJR. It is vital to control the growth rate spent on health care in the USA without compromising patient safety and quality of care. This study shows the ability of two providers to achieve similar cost savings despite geographic and hospital-orthopedic practice integration differences in BPCI program designs, which is paramount to both BPCI and CJR’s nationwide success.

## Electronic supplementary material

ESM 1 (pdf 1.19 MB)

ESM 2 (pdf 1.19 MB)

ESM 3 (pdf 1.19 MB)

ESM 4 (pdf 1.19 MB)

ESM 5 (pdf 1.19 MB)

ESM 6 (pdf 1.19 MB).
